# Impacts of Dietary Patterns and Screen Time on the Risk of Developing Type 2 Diabetes Mellitus Among Children in Saudi Arabia: A Cross-Sectional Study

**DOI:** 10.7759/cureus.107235

**Published:** 2026-04-17

**Authors:** Abdullah A Alotaibi, Ali S Alqarni, Ghadah A Alharbi, Refal H Almutairi

**Affiliations:** 1 Family Medicine, Majmaah University, Al Majma'ah, SAU; 2 Medicine/Operations, Manzil Healthcare Services, Riyadh, SAU; 3 College of Medicine, Majmaah University, Al Majma'ah, SAU

**Keywords:** children, dietary patterns, insulin resistance, screen time, type 2 diabetes mellitus

## Abstract

Background

Type 2 diabetes mellitus (T2DM) is a metabolic disorder characterized by the progressive loss of adequate beta-cell insulin secretion, often occurring in the context of insulin resistance. It is increasingly recognized among children due to changes in lifestyle behaviors. Children’s dietary patterns and prolonged screen time are key modifiable risk factors that may contribute to early metabolic disturbances. In Saudi Arabia, rapid urbanization has led to shifts in eating habits and increased sedentary behaviors among children, warranting further investigation to better understand these risk factors. This study aims to examine the association between dietary patterns, screen time, and early markers of T2DM among children in Saudi Arabia.

Methods

A cross-sectional study was conducted among children using a structured, guardian-reported questionnaire administered electronically in Arabic and English. The questionnaire assessed dietary behaviors, screen time, and early markers of T2DM. Dietary intake was evaluated using a five-point frequency scale (never = 0, always = 5), covering eight food groups. Early markers of T2DM were assessed using a composite score based on five guardian-reported binary items: overweight/obesity diagnosis, rapid weight gain, elevated glucose/insulin levels, diabetes-related symptoms, and family history of T2DM. Statistical analyses were performed using IBM SPSS Statistics for Windows, Version 23.0 (Released 2015; IBM Corp., Armonk, NY, USA), after data cleaning in Microsoft Excel (Microsoft Corp., Redmond, WA, USA).

Results

A total of 292 participants were included in the study. Frequent consumption of unhealthy foods was reported, with 40.8% consuming fast food sometimes and 33.6% often consuming sugar-sweetened beverages. Screen exposure of 30 minutes to 2 hours per day was reported by 29.8% of participants, while 23.6% reported 2-4 hours daily. Overweight or obesity was present in 13.4% of participants, and 15.4% reported recent weight gain. A significant negative correlation was found between dietary behavior scores and T2DM markers (r = -0.257, p < 0.001). Higher screen time was significantly associated with increased T2DM markers (p = 0.001), with the highest scores observed in children who exceeded six hours of screen time daily (1.42 ± 1.06). The combined effect of high screen time and sugary intake was also associated with a higher risk (1.24 ± 1.23 vs. 0.57 ± 0.75, p < 0.001).

Conclusion

This study shows that unhealthy dietary habits and prolonged screen time are significantly associated with early markers of T2DM among children. The combined effect of these behaviors further increases the risk of metabolic syndrome. Promoting healthier eating habits and reducing screen exposure are essential strategies to prevent the early onset of diabetes and improve long-term health outcomes in children.

## Introduction

The prevalence of obesity among children

Type 2 diabetes mellitus (T2DM) is a metabolic disorder characterized by progressive loss of adequate insulin secretion from beta cells [1]. Childhood obesity is a growing global public health concern, with significant implications for long-term health outcomes [2]. The prevalence of obesity varies across countries, with an estimated 1.5-fold increase from 2000-2011 to 2012-2023 [2]. In Saudi Arabia, childhood obesity has become a significant public health issue, reflecting global trends. A large-scale study involving 351,195 children and adolescents reported that approximately 9.4% were classified as obese, while 11.2% were categorized as overweight. The highest obesity rates were observed among children aged 2-6 years, with 12.3% classified as obese. Obesity was also more prevalent in boys (10.4%) than in girls (8.3%) [3]. Additionally, research focusing on school-aged children (6-19 years) found that 21.5% were either overweight or obese, with prevalence increasing with age [4].

The impact of dietary patterns

Globally, poor dietary patterns have emerged as a primary driver of obesity and T2DM among children. The increasing shift toward Western dietary habits, characterized by high consumption of fast food, refined sugars, sugar-sweetened beverages, and processed snacks, has contributed to the rise in childhood obesity and related metabolic disorders. These energy-dense, nutrient-poor diets promote insulin resistance, a key risk factor in the development of T2DM [5]. As these dietary trends become more widespread in both developed and developing countries, the prevalence of T2DM among obese children continues to increase [2]. In Saudi Arabia, these global patterns are not only reflected but have intensified due to rapid urbanization, rising income levels, and increased reliance on convenience foods. Traditional Saudi diets, which are rich in whole grains, dates, and legumes, have increasingly been replaced by energy-dense fast foods, fried items, and sugary drinks. The combined effect of high-calorie diets and sedentary behaviors plays a central role in the rising rates of metabolic syndrome and T2DM among Saudi children [6]. A growing reliance on Western-style diets among Saudi youth has also been reported, with dietary patterns high in processed foods, sugars, and saturated fats becoming more dominant, thereby increasing the risk of obesity and diabetes [7]. Moreover, nutritional assessments of Saudi children have revealed low intake of fruits and vegetables alongside high consumption of snacks and sugar-sweetened beverages, all of which contribute to the early development of insulin resistance and T2DM [8].

The impact of screen time and a sedentary lifestyle

Globally, the rise in screen-based entertainment and digital device use among children has become a significant public health concern, particularly due to its role in increasing the risk of obesity and T2DM. These behaviors are often accompanied by poor dietary habits, such as frequent snacking and the consumption of sugar-sweetened beverages during screen time, which further exacerbate the risk of insulin resistance and metabolic dysfunction, key precursors to T2DM [9]. In Saudi Arabia, these global patterns are especially pronounced due to widespread access to digital technology. High screen time has been significantly associated with unhealthy dietary habits and lower levels of physical activity among Saudi youth, contributing to the increasing prevalence of obesity and metabolic syndrome [10]. A more recent study among Saudi children reported that those who exceeded two hours of daily screen time had significantly higher levels of insulin resistance, waist circumference, and fasting glucose [11].

The risk of type 2 diabetes in obese children

Childhood obesity significantly increases the risk of developing T2DM. The risk is heightened among obese children due to insulin resistance, which is more prevalent in this population [2]. In addition, increased sedentary behaviors and poor dietary habits further contribute to the development of metabolic disorders, including T2DM [3]. A study examining screen time and lifestyle among Australian children found that excessive screen time is directly associated with an increased risk of developing T2DM, particularly among those who are obese [12]. Similarly, research conducted in China reported a higher prevalence of insulin resistance among obese children, a key precursor to T2DM [12]. In the United States, studies have shown that obese children have a higher likelihood of developing T2DM due to the combined effects of genetic predisposition, sedentary lifestyle, and poor dietary intake [6]. In Saudi Arabia, similar findings have been reported, where obesity, along with unhealthy dietary habits, has been shown to increase the risk of T2DM and metabolic syndrome among children [13].

This study aims to examine the association between dietary patterns, screen time, and early markers of T2DM among children in Saudi Arabia. We hypothesize that higher consumption of unhealthy foods (such as fast food, sugar-sweetened beverages, and snacks) and prolonged daily screen time (>2 hours) are independently associated with higher composite T2DM marker scores in this pediatric population.

## Materials and methods

Study design

This was an observational cross-sectional study conducted across the Kingdom of Saudi Arabia. A cross-sectional design was selected for its suitability in assessing the prevalence of exposures and outcomes at a single time point, enabling the identification of associations between lifestyle behaviors and early metabolic risk markers.

Study area

The study covered all regions of the Kingdom of Saudi Arabia. Data were collected between January and March 2026, during the active school term. Regional distribution of participants is provided in the "Results" section.

Study population

The study population included children residing in Saudi Arabia who met the inclusion criteria during the designated data-collection period.

Inclusion criteria

Male and female children aged 2-14 years, currently living in Saudi Arabia, and Arabic or English speakers were included in the study.

Exclusion Criteria

Children younger than two years, older than 14 years, diagnosed with diabetes and genetic syndromes, on steroids or with any chronic disease, living outside Saudi Arabia, and neither Arabic nor English speakers were excluded.

Sampling

A cross-sectional sampling approach was used to collect responses from eligible participants across Saudi Arabia. Due to the wide national distribution, convenience sampling through electronic distribution was applied; the questionnaire was shared via social media platforms and school networks (Appendix 1). Simple random sampling was not feasible given the electronic distribution method; accordingly, readers should note the potential for selection bias inherent to this approach. The required minimum sample size was calculated based on the expected prevalence of childhood overweight/obesity in Saudi Arabia (~21.5%) [4], a margin of error of 5%, and a 95% CI, yielding a minimum required sample of 258 participants. The achieved sample of 292 exceeded this threshold.

Data collection tools and instruments

An electronic questionnaire was distributed among the target participants in both Arabic and English. The questionnaire was developed by the research team and was piloted on a sample of guardians prior to full distribution to assess clarity and face validity. The questionnaire was divided into five sections: (i) demographic information (age of parent, relationship to the child, educational level of parent, child's age, child's gender, region); (ii) dietary patterns, consumption of fruits and vegetables, milk and milk products, whole grains, fast food (e.g., burgers, shawarma, pizza), sugary drinks, sugar-sweetened beverages (e.g., soft drinks, energy drinks, sweetened tea or coffee, flavored milk), traditional Saudi foods, and breakfast skipping. Responses were recorded on a five-point scale (never = 0 to always = 5). Scores for unhealthy food items (fast food, sugary beverages, snacks) were reversed so that higher total dietary behavior scores indicated healthier overall dietary patterns; (iii) screen time and sedentary behavior (mobile/tablet and TV hours, video game use, total screen time, snacking during screen use, and physical activity-related questions). Screen time categories (<30 min, 30 min-2 h, 2-4 h, 4-6 h, >6 h) were defined in reference to WHO guidelines on physical activity and sedentary behavior for children [14]; (iv) early indicators of T2DM, defined as a composite score of five binary guardian-reported items (1, overweight/obesity diagnosis; 2, rapid weight gain in the past year; 3, physician-noted elevated blood glucose or insulin levels; 4, presence of diabetes-related symptoms (fatigue, excessive thirst, or frequent urination); and 5, family history of T2DM). Each item was scored as 0 (no/don't know) or 1 (yes), and the five items were summed to produce a composite T2DM markers score (range 0-5), with higher scores representing a greater number of present risk markers. These items were selected based on established pediatric T2DM risk factor literature [15,16]; and (v) combined lifestyle risk factors (high screen time with high sugary snack intake, rapid weight gain in the past year, screen exposure influencing eating behavior, and overall lifestyle rating).

Ethical approval and informed consent

This study was approved by the Majmaah University Research Ethics Committee (MUREC-Jan.22/COM-2026/316). Informed consent was obtained from all participating guardians prior to questionnaire completion. Guardian consent served as proxy consent for child participation.

Statistical analysis plan

Data were cleaned on Excel (Microsoft Corp., Redmond, WA, USA), extracted, and coded using IBM SPSS Statistics for Windows, Version 23.0 (Released 2015; IBM Corp., Armonk, NY, USA) for analysis. Descriptive statistics were applied to summarize data, synthesize, and report the variables. Numerical data were presented as mean and standard deviation, or as median and interquartile range, according to the type of distribution of each variable. For categorical variables, percentages and frequencies were used. Comparison between groups was made by the Mann-Whitney test and Kruskal-Wallis test, and correlation was performed by the Spearman correlation test. A p-value of less than 0.05 was considered significant. Missing data were handled by complete-case analysis. Potential confounders considered in interpretation included child age, sex, guardian educational level, and region; formal multivariate adjustment was not performed, which is acknowledged as a limitation.

A common scoring system was used for dietary behavior questions (eight questions), in which each response was coded numerically from 0 (never) to 5 (always), and the scores were reversed for unhealthy behaviors (fast food, sugary beverages, and snacks). The total dietary behavior score was calculated by summing the individual item scores, with higher total scores indicating healthier overall dietary patterns.

Early markers of T2DM were assessed using five variables, and each item was scored as 0 (no or don't know) or 1 (yes); then, a composite T2DM marker score was calculated by summing the five items, with higher scores representing a greater number of present risk markers for T2DM.

## Results

A total of 337 questionnaires were distributed to participants, with a response rate of 98.8% (333/337); four participants declined to participate. Forty-one participants were excluded as they did not meet the inclusion criteria, including incomplete responses on key dietary or screening variables, resulting in a final sample of 292 participants. Most of the guardians were 41-50 years old (n = 95, 32.5%), followed by 31-40 years (n = 93, 31.8%). In terms of relationship to the children, the majority of respondents were mothers (n = 212, 72.6%), while 47 (16.1%) were fathers. Nearly two-thirds of the guardians held a bachelor’s degree (n = 190, 65.1%), while 33 (11.3%) had completed postgraduate studies, and 29 (9.9%) had a high school education. The majority of children were 5-7 years old (n = 87, 29.8%), followed by 12-14 years (n = 76, 26.0%). Male children slightly outnumbered females (51.7% vs. 48.3%), with a male-to-female ratio of 1.07:1. Furthermore, more than half of the participants were from the central region (n = 149, 51.0%), while 56 (19.2%) were from the southern region. Further details are presented in Table 1.

**Table 1:** Demographic information of the participants (n = 292) Data are presented as N (%). A p-value < 0.05 was considered statistically significant.

Variable	Categories	N (%)
Age of the guardian	<20 years	5 (1.7)
	20-30 years	63 (21.6)
	31-40 years	93 (31.8)
	41-50	95 (32.5)
	≥51 years	36 (12.3)
Relationship to the child	Mother	212 (72.6)
	Father	47 (16.1)
	Sibling	16 (5.5)
	Other	17 (5.8)
Educational level of the guardian	Elementary	24 (8.2)
	Middle school	16 (5.5)
	High school	29 (9.9)
	Bachelor's degree	190 (65.1)
	Postgraduate studies (master’s/PhD)	33 (11.3)
Child’s age	2-4 years	60 (20.5)
	5-7 years	87 (29.8)
	8-11 years	69 (23.6)
	12-14 years	76 (26)
Child’s gender	Male	151 (51.7)
	Female	141 (48.3)
Region	Central	149 (51)
	Eastern	31 (10.6)
	Western	41 (14)
	Northern	15 (5.1)
	Southern	56 (19.2)

Regarding the dietary behaviors of children, most reported consuming fruits and vegetables sometimes (n = 117, 40.1%), while 105 (36.0%) reported frequent consumption. Milk and dairy products were always consumed daily by 118 (40.4%) of participants’ children, while 90 (30.8%) reported frequent consumption. Concerning whole grain intake, 101 (34.6%) of children sometimes consumed them, while 81 (27.7%) reported always consuming them.

As for unhealthy dietary behaviors, 119 (40.8%) of participants’ children consumed fast food sometimes during a typical week, whereas 74 (25.3%) reported frequent consumption. In addition, 98 (33.6%) children often consumed sugar-sweetened beverages, while 76 (26.0%) sometimes consumed them. Snack consumption was also prevalent, with 100 (34.2%) children sometimes consuming snacks and 95 (32.5%) often consuming them. Conversely, 133 (45.5%) children always ate breakfast daily, and 103 (35.3%) of families regularly consumed three main meals per day (Table 2).

**Table 2:** Dietary patterns among children in Saudi Arabia Data are presented as N (%). A p-value < 0.05 was considered statistically significant. *Grains such as whole wheat, whole-grain bread, oats, brown rice, bulgur, barley, whole corn, and quinoa; fast food such as burgers, shawarma, or pizza; sugar-sweetened beverages such as soft drinks, energy drinks, sweetened tea or coffee, and flavored milk; snacks such as chips, chocolate, biscuits, and candy.

Question	Never	Rarely	Sometimes	Often	Always
	N (%)				
Does your child consume fruits and vegetables daily?	2 (0.7)	23 (7.9)	117 (40.1)	105 (36)	45 (15.4)
Does your child consume milk and dairy products daily?	1 (0.3)	9 (3.1)	74 (25.3)	90 (30.8)	118 (40.4)
Does your child consume whole grains* daily?	7 (2.4)	24 (8.2)	101 (34.6)	79 (27.1)	81 (27.7)
How often does your child have fast food* in a typical week?	10 (3.4)	65 (22.3)	119 (40.8)	74 (25.3)	24 (8.2)
How often does your child drink sugar-sweetened beverages* per day?	24 (8.2)	46 (15.8)	76 (26)	98 (33.6)	48 (16.4)
How many times does your child eat snacks per day?	4 (1.4)	33 (11.3)	100 (34.2)	95 (32.5)	60 (20.5)
Does your child eat breakfast daily?	6 (2.1)	14 (4.8)	55 (18.8)	84 (28.8)	133 (45.5)
How regularly does the family have three main meals a day? (breakfast, lunch, and dinner)	8 (2.7)	18 (6.2)	74 (25.3)	89 (30.5)	103 (35.3)

Regarding screen-related behaviors, the daily screen exposure from mobile phones, tablets, or television among most children ranged from 30 minutes to 2 hours (n = 87, 29.8%), followed by 2-4 hours (n = 69, 23.6%). Only 18 children (6.2%) reported no daily screen use.

For video game use, 81 children (27.7%) reported playing for 30 minutes to 2 hours daily, while 74 (25.3%) reported never using video games, and 65 (22.3%) spent less than 30 minutes per day playing video games.

**Figure 1:**
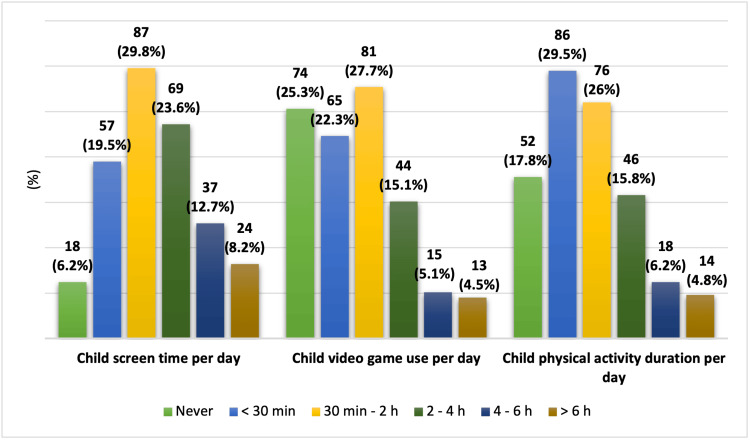
Screen time (TV, mobile devices, gaming) and physical activity among children in Saudi Arabia Data are presented as N (%). A p-value < 0.05 was considered statistically significant.

Regarding physical activity, the most commonly reported duration was less than 30 minutes per day (n = 86, 29.5%), followed by 30 minutes to 2 hours (n = 76, 26.0%). Meanwhile, 52 children (17.8%) reported no daily physical activity (Figure 1).

Regarding early markers of T2DM, the results showed that 39 children (13.4%) were reported as overweight or obese, and rapid weight gain over the past year was observed in 45 (15.4%). Elevated blood glucose or insulin levels during a previous medical visit were reported in only 12 children (4.1%), while 236 (80.8%) were reported not to have such elevations. Moreover, 32 children (11.0%) experienced symptoms suggestive of diabetes, such as fatigue, excessive thirst, or frequent urination, and 108 (37.0%) had a family history of T2DM. Regarding the interaction between screen time and dietary behaviors, the results revealed that the combination of prolonged screen time and high consumption of sugary foods or beverages was reported in more than one-third of children (n = 104, 35.6%). Furthermore, 105 children (36.0%) indicated that their eating habits were affected by screen time, while 132 (45.2%) reported that screen time sometimes affected their eating behaviors (Table 3). It should be noted that all early T2DM markers were based on guardian-reported data and not on objective clinical measurements; therefore, interpretation should be made with appropriate caution.

**Table 3:** Early markers of T2DM, lifestyle factors, and overall lifestyle among children Data are presented as N (%). A p-value < 0.05 was considered statistically significant. T2DM: type 2 diabetes mellitus.

Variable	Categories	N (%)
Has your child been diagnosed as overweight or obese?	Yes	39 (13.4)
	No	253 (86.6)
Have you noticed rapid weight gain in your child over the past year?	Yes	45 (15.4)
	No	247 (84.6)
Has a doctor ever mentioned during a previous visit that your child’s test results indicate elevated blood glucose or insulin levels?	Yes	12 (4.1)
	No	236 (80.8)
	Don't know	44 (15.1)
Does your child experience symptoms such as fatigue, excessive thirst, or frequent urination?	Yes	32 (11.0)
	No	260 (89)
Is there a family history of type 2 diabetes (the type related to insulin resistance and lifestyle factors)?	Yes	108 (37)
	No	144 (49.3)
	Don't know	40 (13.7)
Does your child combine prolonged screen time with consuming large amounts of sugary foods or beverages?	Yes	104 (35.6)
	No	188 (64.4)
Does screen time affect your child’s eating behaviors (such as eating while watching screens or increased appetite)?	Yes	105 (36)
	Sometimes	132 (45.2)
	No	55 (18.8)

**Figure 2:**
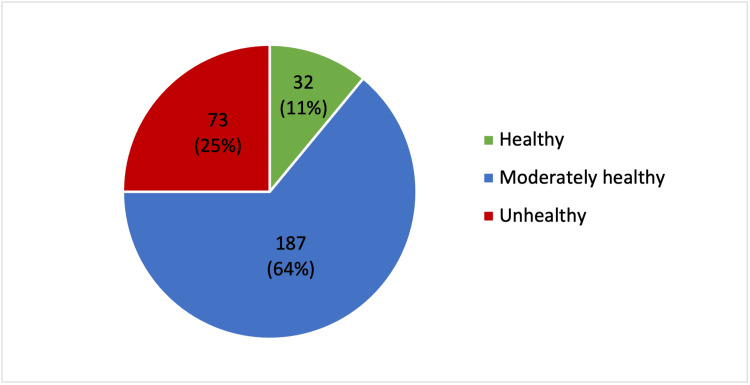
Children’s overall lifestyle rating Data are presented as N (%). A p-value < 0.05 was considered statistically significant.

Concerning the children’s overall lifestyle rating, the majority of participants (n = 187, 64.0%) rated their children’s lifestyle as moderately healthy. Thirty-two (11.0%) rated it as healthy, while a considerable proportion (n = 73, 25.0%) reported that their children had an unhealthy lifestyle (Figure 2).

Regarding the correlation between dietary behavior score and the number of T2DM markers, the results showed a statistically significant negative correlation between dietary behavior score and T2DM markers (Spearman’s rho = -0.257, p < 0.001), indicating that better dietary behaviors were associated with fewer early markers of T2DM among children (Table 4).

**Table 4:** Correlation between dietary behavior score and number of T2DM markers Data are presented as Spearman’s rho correlation coefficient. A p-value < 0.05 was considered statistically significant. T2DM: type 2 diabetes mellitus.

Variable	Spearman’s rho correlation coefficient	p-value
Dietary behavior score and T2DM markers score	-0.257	<0.001

Regarding the association between lifestyle factors and early markers of T2DM, the results demonstrated a statistically significant association between the duration of daily use of mobile phones, tablets, or television and T2DM marker scores (H(5) = 19.68, p = 0.001). Children who spent more than six hours per day using mobile phones, tablets, or watching television had the highest T2DM marker scores, while those who spent less than 30 minutes per day had the lowest (1.42 ± 1.06 vs. 0.63 ± 1.06).

Similarly, a significantly higher T2DM marker score was observed among children who combined prolonged screen time with high consumption of sugary foods or beverages compared with those who did not engage in both behaviors (1.24 ± 1.23 vs. 0.57 ± 0.75; U = 6611.5, p < 0.001).

Moreover, screen time affecting a child’s eating behaviors was significantly associated with higher T2DM marker scores (H(2) = 15.15, p = 0.001). Children whose eating behaviors were affected had the highest mean scores compared with those whose eating behaviors were sometimes affected or not affected at all.

Other factors, including duration of daily video game use and physical activity, were not significantly associated with T2DM marker scores (p > 0.05) (Table 5).

**Table 5:** The relationship between lifestyle factors and early markers of T2DM Data are presented as mean (SD). A p-value < 0.05 was considered statistically significant. ^€^Kruskal-Wallis test. ^£^Mann-Whitney U test.

Variable	Categories	T2DM marker score	Test statistic	p-value
		Mean (SD)		
How many hours does your child use a mobile phone, iPad/tablet, or watch television per day?	Never	1.11 (1.37)	H(5) = 19.678	0.001^€^
	<30 min	0.63 (1.06)		
	30 min to 2 h	0.64 (0.85)		
	2-4 h	0.70 (0.77)		
	4-6 h	1.14 (1.16)		
	>6 h	1.42 (1.06)		
How many hours does your child play video games per day? (PlayStation, Xbox, computer)	Never	0.81 (1.06)	H(5) = 8.125	0.149^€^
	<30 min	0.66 (0.96)		
	30 min to 2 h	0.72 (0.97)		
	2-4 h	1.02 (0.93)		
	4-6 h	1.20 (1.27)		
	>6 h	0.92 (0.95)		
How much time does your child spend in physical activity each day?	Never	1.02 (1.15)	H(5) = 7.166	0.209^€^
	<30 min	0.98 (1.21)		
	30 min to 2 h	0.55 (0.74)		
	2-4 h	0.74 (0.80)		
	4-6 h	0.61 (0.70)		
	>6 h	0.86 (0.95)		
Does your child combine prolonged screen time with consuming large amounts of sugary foods or beverages?	Yes	1.24 (1.23)	U = 6611.5	< 0.001^£^
	No	0.57 (0.75)		
Does screen time affect your child’s eating behaviors (such as eating while watching screens or increased appetite)?	Yes	1.15 (1.22)	H(2) = 15.149	0.001^€^
	Sometimes	0.62 (0.78)		
	No	0.60 (0.85)		

## Discussion

T2DM is increasingly emerging among children, largely driven by lifestyle and behavioral changes [15]. Unhealthy dietary patterns, including high consumption of energy-dense foods and sugar-sweetened beverages, along with prolonged screen time, contribute to obesity and metabolic disturbances [16]. These factors often coexist and can promote early insulin resistance and impaired glucose regulation. In Saudi Arabia, rapid urbanization and lifestyle changes have intensified these risks, highlighting the need to examine how diet and screen exposure jointly affect early markers of diabetes and metabolic syndrome in children [17,18]. This study aimed to explore the relationship between dietary patterns, screen time, and early markers of T2DM among children in Saudi Arabia.

The demographic profile showed that most respondents were mothers and were highly educated. The children were fairly evenly distributed across age groups, with a slight predominance of males. Regarding dietary habits, although a reasonable proportion of children frequently consumed fruits, vegetables, and dairy products, the simultaneous high intake of unhealthy foods is notable. Similarly, Alsubaie et al. reported a high prevalence of low consumption of fruits, vegetables, and dairy products among children in Saudi Arabia [19]. In the present study, a large proportion of children consumed fast food, sugar-sweetened beverages, and snacks regularly. This dual pattern, partial adherence to healthy foods alongside frequent unhealthy consumption, reflects a mixed dietary pattern, which has been described in rapidly urbanizing societies. Consistent with this, Mumena et al. reported increased consumption of energy-dense, nutrient-poor foods among Saudi children, influenced by lifestyle changes, easy access to fast food, and marketing exposure [20].

The high intake of sugar-sweetened beverages observed in this study is particularly concerning. Previous research has shown that these beverages are strongly associated with insulin resistance, obesity, and an increased risk of T2DM in children. Calcaterra et al. reported that frequent consumption of sugary drinks leads to excess caloric intake and impaired glucose metabolism, thereby increasing the risk of metabolic syndrome and diabetes in children [21]. Similarly, frequent snacking and fast food consumption have been linked to poor glycemic control and increased adiposity in pediatric populations.

Screen time behaviors in this study showed that a substantial proportion of children spent between 30 minutes and 4 hours daily on screens, with a smaller but important group exceeding 6 hours. These findings are consistent with Bhor et al., who reported that increased screen time is significantly associated with elevated cardiometabolic risk among children and adolescents, with each additional hour contributing to higher risk [22]. Despite international guidelines recommending limited recreational screen time, adherence remains low [23].

One of the key findings of this study is the significant association between screen time duration and T2DM markers. Given the cross-sectional design, these findings indicate associations rather than causation. Children with higher screen exposure had significantly higher T2DM marker scores, with the highest levels observed in those exceeding six hours per day. This is consistent with findings by Franssen et al., who reported that prolonged sedentary behavior contributes to metabolic dysfunction and diabetes risk [24]. This may be explained by reduced physical activity, increased unhealthy eating behaviors, and greater adiposity associated with prolonged screen use.

Interestingly, the duration of video game use and physical activity was not significantly associated with T2DM markers in this study. This contrasts with existing literature that typically demonstrate protective effects of physical activity. Possible explanations include measurement limitations, reliance on self-reported data, variability in activity intensity, or insufficient duration of physical activity to yield metabolic benefits. It may also suggest that sedentary screen exposure independently contributes to metabolic risk beyond physical inactivity alone.

A key observation of this study is the interaction between screen time and dietary behavior. More than one-third of children combined prolonged screen time with high consumption of sugary foods or beverages, and this combination was associated with higher T2DM marker scores. This relationship should be interpreted as correlational rather than causal. These findings support the concept of behavioral clustering, in which multiple unhealthy habits coexist and amplify disease risk. Previous studies have shown that children exposed to screens are more likely to engage in mindless eating, increased caloric intake, and a preference for high-sugar foods, often influenced by food advertising [25]. In addition, this study found that screen time significantly affected children’s eating behaviors, with those reporting such effects having higher T2DM marker scores. This aligns with evidence suggesting that eating while viewing screens disrupts satiety signals and promotes overeating, highlighting the role of environmental and behavioral cues in shaping dietary habits [26,27].

Correlation analysis in this study also revealed a significant negative association between dietary behavior scores and T2DM markers, indicating that healthier dietary patterns are linked to lower metabolic risk. Although the correlation was modest (rho = -0.257), it remains clinically relevant, particularly in pediatric populations where early interventions can yield long-term benefits. However, this finding should not be overstated, and the absence of multivariate analysis limits the ability to account for potential confounders such as age, sex, and socioeconomic status. These findings are consistent with previous research showing that adherence to healthy dietary patterns, such as higher intake of fruits, vegetables, and whole grains, is associated with improved insulin sensitivity and reduced diabetes risk [28].

Regarding early T2DM markers, the prevalence of overweight/obesity (n = 39, 13.4%), rapid weight gain (n = 45, 15.4%), and family history (n = 108, 37.0%) indicates a population already at risk. Although only a small proportion of participants reported elevated glucose or insulin levels, this may reflect underdiagnosis or limited routine screening in children. The presence of diabetes-related symptoms in 32 children (11.0%) further underscores the importance of early detection. It is important to note that all early T2DM markers in this study were based on guardian-reported data rather than objective clinical measurements; therefore, these findings should be interpreted with caution.

Limitations

This study has several limitations. The cross-sectional design limits the ability to infer causality between lifestyle factors and the risk of T2DM among children. All data were guardian-reported, which may introduce recall and reporting bias. Social desirability bias may also have led to underreporting of unhealthy dietary behaviors, potentially attenuating the observed associations. In addition, the accuracy of guardian-reported dietary data may vary depending on the child’s age and the guardian’s educational level. The use of subjective measures for dietary intake and screen time may further reduce the accuracy of the findings. Moreover, the sample may not fully represent all regions or socioeconomic groups in Saudi Arabia; therefore, the findings are most applicable to families with access to digital platforms, and caution is needed when generalizing the results. The lack of objective clinical measurements may have resulted in the underestimation of early metabolic abnormalities. Finally, the unadjusted statistical analyses limit the ability to control for potential confounders such as age, sex, and socioeconomic status.

Implications and future directions

There is a need for early preventive strategies that target both dietary habits and screen time among children. Family-based and school-centered interventions should promote healthy eating behaviors and reduce sedentary activities. Future research should adopt longitudinal designs and incorporate objective clinical measures to better assess causality. In addition, exploring culturally tailored interventions and policy-level actions in Saudi Arabia will be essential to effectively reduce the rising risk of T2DM among children.

## Conclusions

Our study shows that unhealthy dietary patterns and prolonged screen time are significantly associated with early markers of T2DM among children in Saudi Arabia. Children exposed to higher screen time and poor dietary habits demonstrated a greater risk of metabolic disturbances, particularly when these behaviors coexisted. Healthier dietary practices were associated with a lower risk of diabetes. Given the cross-sectional design, these findings reflect associations and should not be interpreted as evidence of causation. Overall, these results highlight the importance of addressing modifiable lifestyle factors early to prevent the progression of metabolic disorders and reduce the future burden of T2DM.

## Appendices

Appendix 1 

**Table 6:** Questionnaire assessing demographic characteristics, dietary patterns, screen time, physical activity, and risk factors for type 2 diabetes among children

Section	Question	Options
Section 1: demographic information	Age of the guardian	20 years or less / 21-30 years / 31-40 years / 41-50 years / 51+ years
	Relationship to the child	Mother / Father / Relative (specify)
	Educational level	Primary / Intermediate / Secondary / Bachelor’s / Postgraduate
	Child age	2-5 years / 6-10 years / 11-14 years
	Child gender	Male / Female
	Region	Central / Western / Eastern / Southern / Northern
Section 2: dietary patterns	Fruits and vegetables (daily)	None / 1 / 2 / 3+
	Milk and dairy (daily)	None / 1 / 2 / 3+
	Whole grains (daily)	None / 1 / 2 / 3+
	Fast food (weekly)	Never / Once / 2-3 times / 4+ times
	Sugary drinks (daily)	None / 1 / 2 / 3+
	Snacks (daily)	Rarely / 1-2 / 3-4 / 4+
	Breakfast habit	Yes / No
	Family meals regularity	Always / Often / Sometimes / Rarely / Never
Section 3: screen time and physical activity	Screen time per day	<30 min / 30 min-2 h / 2-4 h / 4+ h
	Video games per day	None / <30 min / 30 min-2 h / 2+ h
	Physical activity per day	<30 min / 30 min-2 h / 2-4 h / 4+ h
Section 4: early indicators of type 2 diabetes	Overweight/obese diagnosis	Yes / No
	High glucose/insulin signs	Yes / No / Not sure
	Symptoms (fatigue, thirst, urination)	Yes / Sometimes / No
	Family history of diabetes	Yes / No
Section 5: combined risk factors	Screen time + sugary intake	Yes / No
	Rapid weight gain	Yes / No
	Screen affecting eating behavior	Yes / Sometimes / No
	Overall lifestyle	Healthy / Moderately healthy / Unhealthy
